# Genomic Structural Equation Modelling Reveals the Shared Genetic Architecture for Oral Frailty

**DOI:** 10.3290/j.ohpd.c_2329

**Published:** 2025-11-26

**Authors:** Yan Chen, Ling Wang, Tingyan Zeng

**Affiliations:** a Yan Chen Researcher, Department of Stomatology, The Third Hospital of Changsha (The Affiliated Changsha Hospital of Hunan University), Changsha, China. Conceived and designed the study, conducted the analysis and wrote the paper.; b Ling Wang Researcher, Department of Stomatology, The Third Hospital of Changsha (The Affiliated Changsha Hospital of Hunan University), Changsha, China. Conducted the analysis and wrote the paper.; c Tingyan Zeng Researcher, Department of Stomatology, The Third Hospital of Changsha (The Affiliated Changsha Hospital of Hunan University), Changsha, China. Conceived and designed the study.

**Keywords:** fine-mapping, GsMap, genomic SEM, oral frailty, post-GWAS

## Abstract

**Purpose:**

Oral frailty, an age-related decline in oral function and health linked to adverse geriatric outcomes, involves multiple phenotypes. Chronic periodontitis, a key inflammatory driver of tooth loss and systemic disease, is a cornerstone of this syndrome, yet the shared genetic architecture connecting it to other oral conditions remains uncharacterised.

**Methods and Materials:**

We employed genomic structural equation modelling (genomic SEM) to integrate genome-wide association studies (GWAS) summary statistics from five oral frailty-related phenotypes, defining a common latent factor reflecting their shared genetics. We further integrated several post-GWAS analytical methods, including locus and gene discovery (MAGMA, TWAS/FOCUS), fine-mapping (SuSiE, FINEMAP), pathway and cell-type enrichment (S-LDSC, CELLECT), spatial mapping (gsMap), and Polygenic Risk Score analyses.

**Results:**

The genomic SEM model demonstrated a good fit and revealed a common genetic factor underlying oral frailty. We identified four genome-wide significant loci, three of which are novel for oral frailty. Fine-mapping prioritised rs150699482 (KIAA0247), rs78975199 (SPG11), and rs2705755 (SNORA77) as likely causal variants. MAGMA highlighted 13 candidate susceptibility genes, with SPG11 and CCDC91 among the top candidates. TWAS and FOCUS analyses robustly implicated RP11-967K21.1 as a putative causal gene. Cell-type enrichment analysis indicated significant involvement of brain endothelial cells, immune cells, and mammary gland stromal cells. Heritability was enriched in evolutionarily conserved regions and active regulatory elements. Notably, gsMap analysis showed that genetic risk for oral frailty is enriched not only in jaw and tooth tissues but also across multiple embryonic tissues, including adipose tissue, dorsal root ganglion, mucosal epithelium, and connective tissue.

**Conclusions:**

This study provides the first comprehensive genomic SEM-based characterisation of the shared architecture underlying oral frailty-related traits. By demonstrating a common genetic basis linking periodontitis with other oral dysfunctions, it provides novel insights into shared aetiological pathways and reinforces the concept of oral frailty as a systemic issue.

Oral frailty has emerged as a critical dual-purpose health marker, simultaneously reflecting functional vitality and serving as an early indicator of senescence. This syndrome encompasses a spectrum of complex conditions that compromise oral structures and functions, with a particularly high prevalence among older adults.^12^ Central to this decline is chronic periodontitis, a highly prevalent inflammatory disease that leads to the progressive destruction of tooth-supporting tissues, resulting in tooth mobility and eventual tooth loss.^13^ The chronic inflammatory burden imposed by periodontitis is a major public health concern, as a substantial body of epidemiological evidence now links it to an increased risk for numerous systemic diseases, including several types of cancer.^18,58^ Consequently, managing oral health, with a strong focus on controlling periodontal inflammation, is a cornerstone of healthy ageing strategies.^11^ However, the global trend of population ageing is projected to exacerbate the burden of age-associated oral diseases,^37^ underscoring the urgent need to elucidate their underlying pathogenesis and to develop effective interventional strategies. The aetiology of these oral frailty-related phenotypes is multifactorial, arising from intricate interactions among genetic predispositions, environmental exposures, age-related physiological changes, and systemic health conditions.^39^ Importantly, they frequently co-manifest, suggesting shared pathogenic mechanisms. The mechanistic links between periodontitis and systemic disease are becoming clearer, involving persistent microbial dysbiosis, the systemic dissemination of oral pathogens, and a failure to resolve chronic inflammation, all of which can contribute to both local tissue destruction and systemic pathology.^5,52,65^ For instance, chronic periodontitis leads to the degradation of periodontal structures, resulting in tooth mobility, which in turn impairs mastication and adversely affects nutritional status.^26,45^ Similarly, Sjögren’s syndrome has been implicated in increased susceptibility to multiple oral diseases and in the development of dysphagia 56. These observations highlight the importance of identifying common underlying mechanisms that drive the complex interplay among these conditions. Although epidemiological studies have increasingly revealed associations among diverse oral diseases,^37^ the shared aetiological basis – particularly the role of common genetic factors – remains largely unexplored. This knowledge gap represents a significant barrier to the development of targeted preventive and therapeutic interventions aimed at mitigating age-related oral functional decline.

Historically, research has focused on individual oral conditions. Genetic studies, for example, have successfully identified several susceptibility genes for periodontitis.^16^ However, such approaches cannot determine whether the same genetic factors also increase risk for other co-occurring oral problems. The intricate nature of the immune response in periodontal disease, involving complex pathways such as the PD-1/PD-L1 immune checkpoint, which also plays a role in cancer immune evasion, underscores the need for unbiased approaches that can capture this pleiotropic complexity.^16^ As a result, findings from these studies fail to capture the complexity of syndromes such as oral frailty. Thus, there is a pressing need for more comprehensive and unbiased approaches to unravel the genetic architecture of these conditions.

The advent of genome-wide association studies (GWAS) has revolutionised the field by enabling systematic, unbiased scanning of genetic variants across the genome.^3,34^ GWAS has been successfully applied to investigate the genetic basis of several oral conditions, including Sjögren’s syndrome, chronic periodontitis, and dental caries.^9,25^ However, these studies have typically analysed each phenotype in isolation, thereby failing to determine whether diverse oral phenotypes share common genetic pathways.^11^ This limitation has hindered a comprehensive understanding of why periodontal disease often co-occurs with other signs of oral decline, underscoring the need for advanced statistical genetic methodologies.

To overcome these challenges, the present study was designed to test the central hypothesis that diverse oral frailty-related phenotypes – specifically Sjögren’s syndrome, periodontitis, dental caries, dysphagia, and loose teeth – share a common genetic architecture that contributes to a general liability for age-related oral functional decline. We hypothesised that this shared genetic basis could be statistically as a latent common factor. To this end, we employ genomic structural equation modelling (Genomic SEM) to: (1) estimate the genetic covariance structure among these five traits using GWAS summary statistics; (2) identify and validate a latent genetic factor representing oral frailty; (3) conduct a GWAS on this latent factor to discover novel genetic loci associated with this shared vulnerability; and (4) use a suite of post-GWAS analyses to translate these genetic findings into plausible biological mechanisms. This integrative approach offers a systematic, hypothesis-driven perspective on the shared genetic underpinnings of oral frailty, ultimately informing the development of preventive strategies and targeted interventions for at-risk populations.

## METHODS AND MATERIALS

### Overall Framework and Analytical Workflow

**Fig 1:**
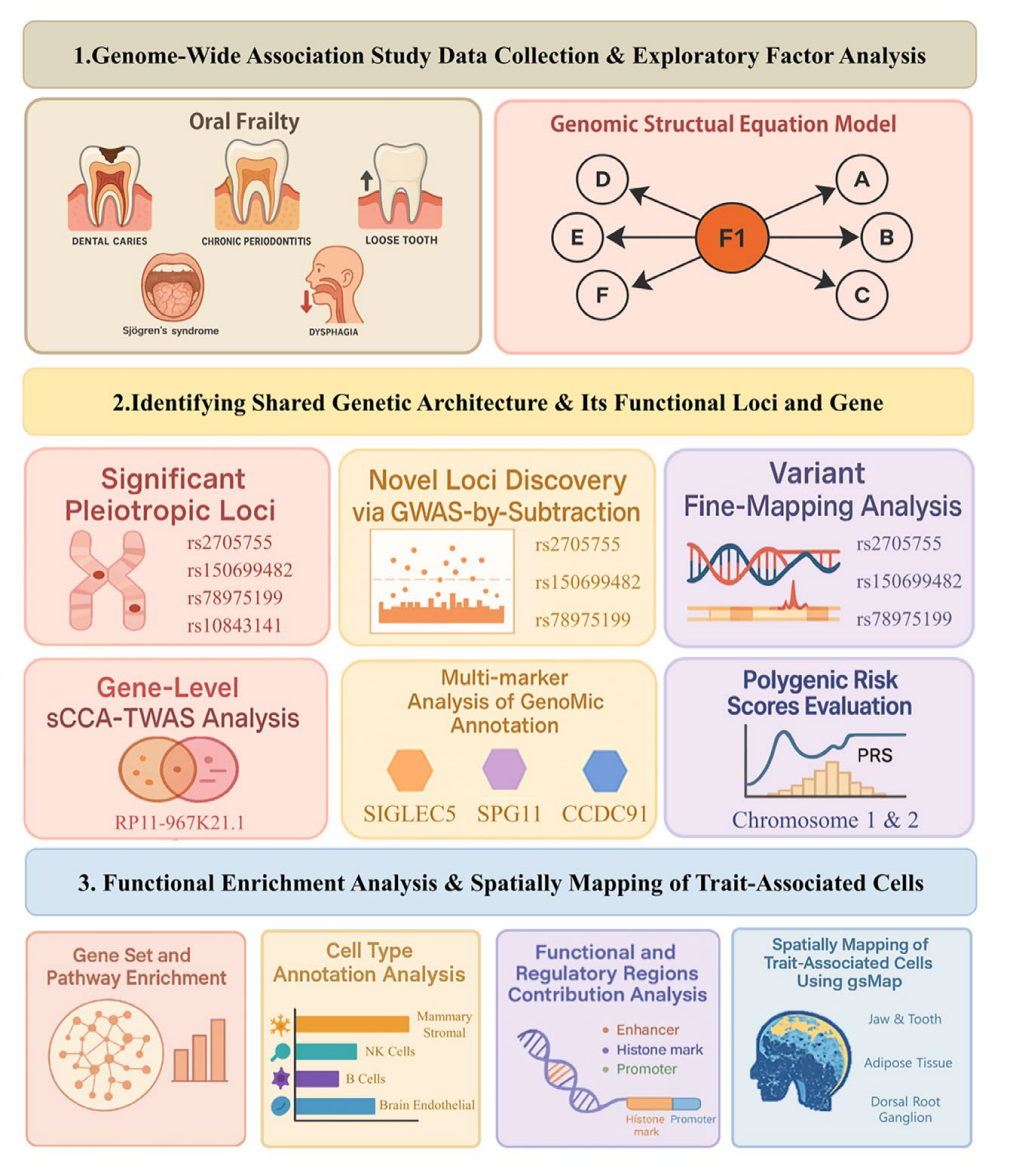
Flowchart illustration.

Figure 1 provides a schematic overview of the workflow adopted in this study to elucidate the genetic architecture of oral frailty. Initially, GWAS summary statistics for five oral frailty-related phenotypes were collected and subjected to rigorous quality control, thereby establishing a robust foundation for subsequent analyses. Genomic SEM was then applied to the QC’ed data, which enabled the identification of a latent common factor underlying oral frailty. To validate the model and its results, SNP heterogeneity testing and genomic control were performed. Subsequently, efforts were directed toward identifying novel risk loci for oral frailty by comparing novel loci with previous input GWAS, utilising FUMA and the GWAS-minus-locus method. Potential pleiotropy was further examined through the GWAS catalogue. Fine-mapping approaches, including SuSiE and FINEMAP, were employed to pinpoint likely causal variants within these risk loci. To link genetic variants to their corresponding biological functions, causal gene inference was conducted. A cross-tissue transcriptome-wide association study based on sparse canonical correlation analysis (sCCA-TWAS) using FUSION identified genes whose predicted expression was associated with oral frailty risk, while FOCUS was used to identify likely causal genes. Additionally, MAGMA was utilised to map SNP-level information to the gene level, which enabled subsequent gene-set enrichment analyses. The biological relevance of the genetic findings was further explored through enrichment analyses. Pathway enrichment was performed using gene sets identified by MAGMA, and cell-type enrichment was assessed with CELLECT and Tabula Muris scRNA-seq data. Heritability partitioning across genomic regions was conducted using stratified Linkage Disequilibrium Score Regression (S-LDSC), which enabled the identification of key functional elements. Finally, to spatially map cells associated with oral frailty and clarify their tissue context, the gsMap pipeline was employed. Polygenic risk scores (PRS) were constructed using PRS-CS to quantify individual genetic predisposition.

### GWAS Summary Statistics Data Sources for Genomic SEM

To establish a robust genetic foundation, this study utilised publicly available GWAS summary statistics for five phenotypes relevant to oral frailty: Sjögren’s syndrome (FinnGen R11, cases/controls = 2,981/439,424), chronic periodontitis (FinnGen R11, 5,364/288,472), loose teeth^23^ (18,545/436,020), dental caries^64^ (3,051/398,136), and dysphagia^64^ (6,482/369,275). All contributing studies obtained ethical approval and informed consent. Table S1 details the incorporated GWAS data sets used here. The unifying hypothesis is not that these conditions share identical initiating causes, but that they partly reflect a common, age-sensitive genetic susceptibility rooted in core biological systems, including chronic inflammation, immune regulation, tissue remodelling and repair, barrier integrity, and neuromuscular function, which together determine an individual’s resilience to diverse insults.^48^ For instance, while periodontitis and Sjögren’s syndrome arise from distinct microbial and autoimmune triggers, respectively, they both converge on pathways of immune dysregulation and tissue-damaging inflammation, with evidence for overlapping susceptibility genes and shared signalling axes.^59,60^ Furthermore, host–microbiome interplay provides another mechanistic route for pleiotropy, as host genetic variation shapes oral microbial community states and modulates responses to biofilms, thereby influencing risk for both caries and periodontal disease.^6,32^ In addition, autoimmune glandular injury in Sjögren’s syndrome secondarily increases susceptibility to caries and swallowing difficulties,^57^ and systemic processes like sarcopenia link dysphagia with broader oral health via shared neuromuscular and metabolic pathways.^49^ Within this framework, we defined chronic periodontitis and loose teeth as related but distinct phenotypes to capture complementary aspects of the disease. Periodontitis represents the inflammatory disease process, while loose teeth index the functional endpoint of cumulative structural damage. This separation allows the model to absorb their shared genetic liability while retaining trait-specific genetic variance related to distinct processes like immune signalling versus bone metabolism and tissue integrity.^55^

### Quality Control of Input GWAS Data

A stringent QC pipeline was implemented, filtering autosomal SNPs using the 1000 Genomes Project Phase 3 European reference panel. Variants with minor allele frequency (MAF) <0.01, inconsistent or ambiguous alleles, or located within the major histocompatibility complex (MHC) region (chr6: 25–35 Mb) were excluded to minimise bias. Given that our analysis incorporated GWAS summary statistics from multiple cohorts, the potential for participant overlap presented a significant methodological challenge. We addressed this by implementing the multivariate extension of LDSC within the genomic SEM paradigm. This statistical framework is specifically designed to estimate the matrix of genetic covariances while simultaneously deriving a correction for any sample overlap reflected in the input data. The primary objectives of this step were to preclude spurious inflation of test statistics and to fortify the validity of subsequent genomic SEM findings by minimising bias in effect size parameters.

### Genomic SEM Construction

Genomic SEM was performed using the Genomic-SEM R package (v0.0.5), enabling investigation of latent genetic structures across the five traits. The analysis involved two stages 17. The first stage estimated the empirical genetic covariance matrix and its corresponding sampling covariance matrix. QC-filtered GWAS summary statistics were compiled for this purpose. Then, the multivariate LDSC generated the empirical genetic covariance matrix for the five traits, which served as the essential input for the SEM model fitting in the subsequent stage. SNP-based heritability (h^2^SNP) estimates from LDSC are reported (Table S2). The second stage involved fitting a common factor SEM model to identify a latent common genetic factor underlying the five input traits. It was achieved by minimising the discrepancy between the model-implied covariance structure and the empirical genetic covariance matrix derived from Stage 1. Model adequacy was evaluated using indices such as standardised root mean square residual (SRMR), model Chi-square (χ^2^), Akaike information criterion (AIC), and comparative fit index (CFI) (Tables S3 and S4). By implementing an appropriate common factor SEM, we integrated association information from individual autosomal SNPs into the genetic and sample covariance matrices, enabling genome-wide analysis of shared covariance across the oral frailty GWAS data sets. To ensure consistent effect directions, we performed a heterogeneity test for each SNP, excluding those with a Cochran’s Q statistic P value <0.05.

### Multi-level Evaluation of the Genomic SEM Model

Beyond standard fit indices, we used an alternative LDSC approach to assess the stability and validity of the genomic SEM model. The model was assessed using parameters including mean χ^2^, Lambda GC, maximum χ^2^, h^2^, intercept value, and the ratio (calculated as (LDSC Intercept – 1) / (Mean χ^2^ – 1)). Control parameters included retaining SNPs with missing values, INFO scores <0.9, MAF <0.01, and excluding SNPs with invalid P values or ambiguous strand direction.

### Identification of Significant and Novel Genomic Loci

We used the FUMA platform to identify significant SNPs (P <5 × 10^–8^), lead SNPs (r^2^ <0.1).^21^ We also employed a ‘GWAS-minus-locus’ approach to ascertain novelty. A locus identified by genomic SEM was operationally defined as ‘novel’ if its lead SNP was located >1 Mb from any SNP previously reported at genome-wide significance (P <5 × 10^–8^) in the single-trait GWAS or prior literature. Further comparisons were made using previously published GWAS catalogue associations. Gene-based and gene-set enrichment analyses were performed using MAGMA. Significance was determined using an FDR <0.05.

### Fine-mapping of Association Signals

We implemented SuSiE and FINEMAP using the echolocatoR R package (v2.0.3) to pinpoint likely causal SNP1we present a novel method, Finemap-MiXeR, for finemapping causal variants from GWAS summary statistics, controlling for correlation among variants due to linkage disequilibrium. Our method is based on a variational Bayesian approach and direct optimization of the Evidence Lower Bound (ELBO. A 250 kb window centred on each lead SNP was analysed, and posterior probabilities (PP) of causality were calculated. Variants achieved mean PP >0.95 within 95% credible sets were prioritised as the most likely causal SNP.

### Transcriptome-wide Association Study

Given that association signals may be mediated through gene expression levels and fine-mapping based solely on SNP proximity can be limited. SCCA-TWAS was conducted to identify genes whose genetically predicted expression levels are associated with oral frailty.^33^ It leveraged pre-computed tissue-specific expression quantitative trait locus (eQTL) weights, covering 36,149 filtered gene features from GTEx (v8). Genes with significant TWAS association (FDR <0.05) were further refined using FOCUS, which calculates the posterior inclusion probability (PIP) for each gene. This Bayesian approach integrates GWAS summary statistics and eQTL weights, carefully adjusting for complex LD structure among SNPs and the predicted expression levels of multiple genes within the locus, while also accounting for potential colocalisation effects. A PIP >0.8 identified likely causal genes.

### Gene-Set and Pathway Enrichment Analysis

Gene-set and pathway enrichment analyses elucidated biological functions of oral frailty, which used the molecular signatures database (MsigDB) with gene-set enrichment analysis (GSEA), focusing on genes identified by previous MAGMA analysis. Enrichment was assessed against the background of all protein-coding genes. Significance was determined using an FDR <0.05.

### Cell-Type Annotation Analysis and Regional Contribution Analysis

We performed cell-type-specific enrichment analysis using CELLECT, which identified cell types linked to oral frailty.^53^ GWAS summary statistics were integrated with scRNA-seq data from the Tabula Muris mouse database, which includes 100,000 cells across 20 tissues.^47^ CELLECT tested for heritability enrichment using CELLEX preprocessed data, applying S-LDSC to determine if oral frailty heritability was significantly concentrated in genes specifically expressed within particular cell types. S-LDSC with the baseline-LD model was also used to partition heritability across broader functional genomic annotations.^41^ Significance was determined using an FDR <0.05.

### Spatially Resolved Mapping of Trait-Associated Cells Using gsMap

To spatially map cells associated with the oral frailty and elucidate their tissue context, we used the gsMap, integrating mouse embryonic spatial transcriptomics data (E16.5_E1S1.MOSTA) with oral frailty GWAS summary statistics.^50^ To enable cross-species analysis, a mouse–human gene homologue mapping file was utilised.

### Polygenic Risk Score Construction and Evaluation

PRS were constructed using the PRS-CS algorithm, which estimated posterior effect sizes for SNPs by integrating GWAS summary statistics with LD information from an external reference panel. Posterior SNP effect size estimates were used to calculate individual-level PRS scores.^14^

## RESULTS

### Structural Equation Model Fitting

**Fig 2:**
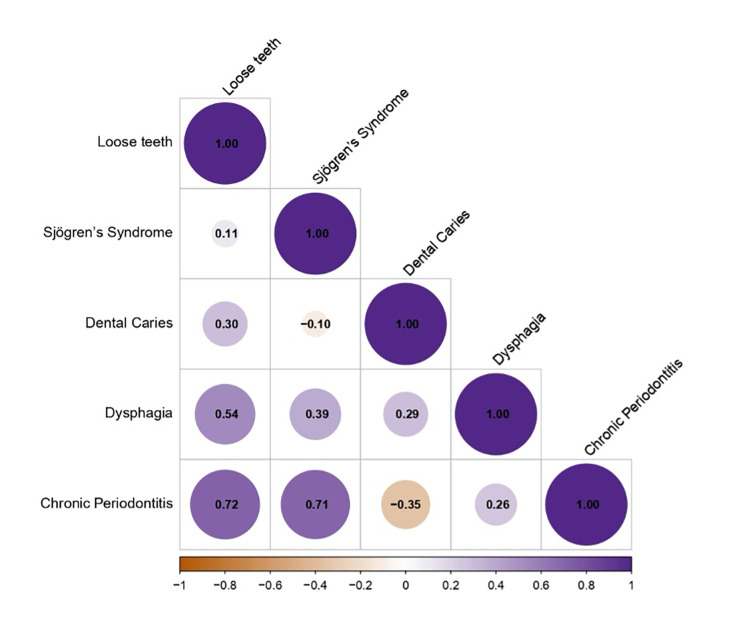
Genetic correlation matrix of oral frailty. The colour intensity and circle size represent correlation strength, ranging from − 1 to + 1.

Analysis of five input GWAS traits using LDSC indicated significant heritability (h^2^ Z-score >1.96) for four phenotypes, with caries as the exception (h^2^ Z-score = 0.932; Table S2). Genetic covariances between trait pairs are presented in Table S3 and Figure 2. A single common factor model fit the empirical covariance matrix well (CFI = 0.849; Table S4). Furthermore, a sensitivity analysis excluding dental caries confirmed the model’s robustness (CFI = 0.845). Standardised factor loadings and residual variances are provided in Table S5. Genomic SEM analysis generated GWAS summary statistics for oral frailty.

### Stability Assessment of the Genomic SEM via LDSC

After QC, 1,121,132 high-quality SNPs were retained. The mean χ^2^ statistic was 1.242, Lambda GC was 1.297, and the maximum χ^2^ was 29.757. LDSC estimated h^2^ SNP for oral frailty at 0.088 (SE = 0.009), with an intercept of 1.0954 (SE = 0.0083) and attenuation ratio of 0.3938 (SE = 0.0341), indicating that observed genomic inflation is primarily attributable to polygenicity.

### Risk Genetic Loci

**Fig 3:**
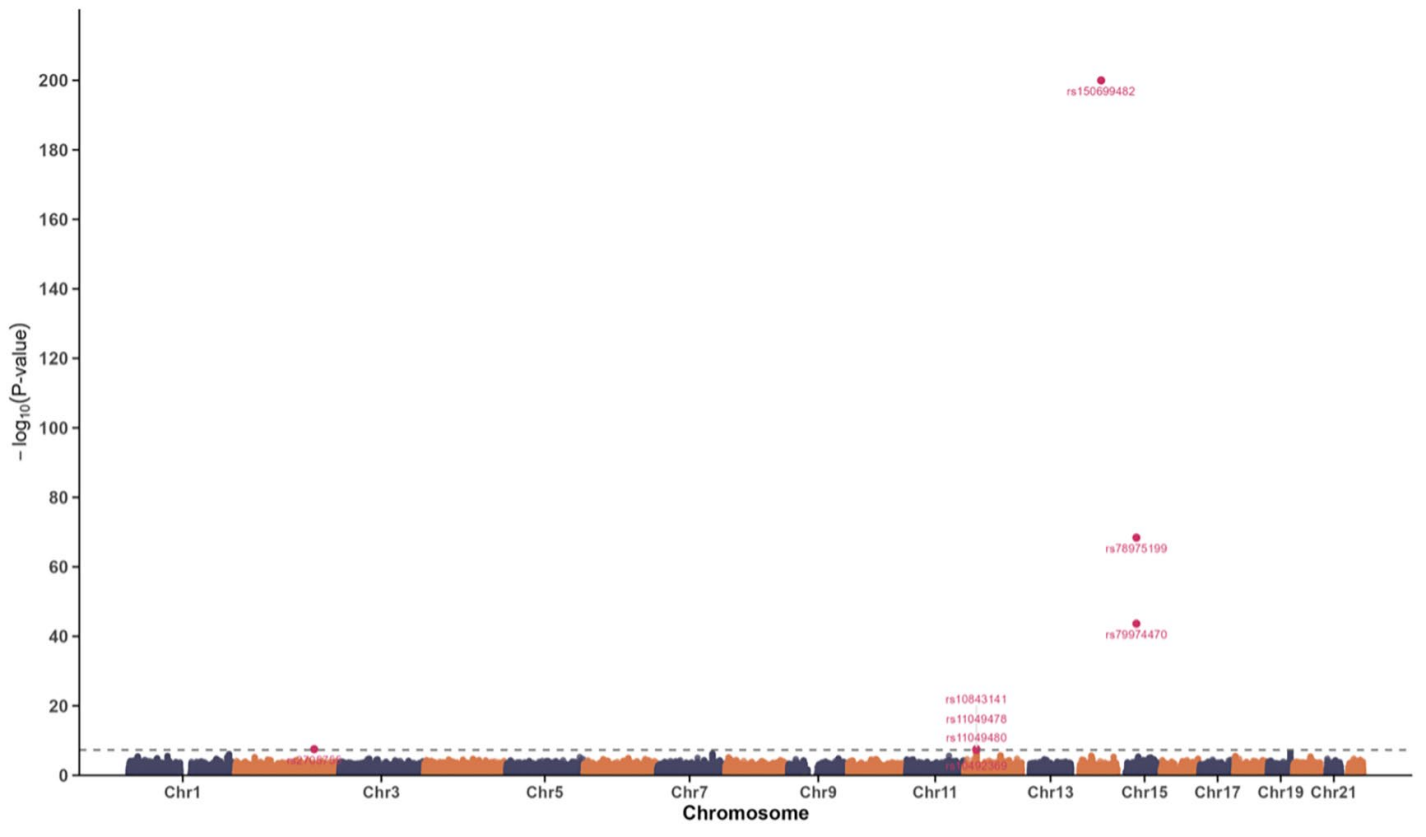
Manhattan plot of novel genomic-SEM results for oral frailty. The x-axis denotes chromosomal positions, while the y-axis represents the negative logarithm of the P-value (− log10(P)). The dashed line indicates the genome-wide significance threshold at P = 5 × 10^−8^.

**Fig 4:**
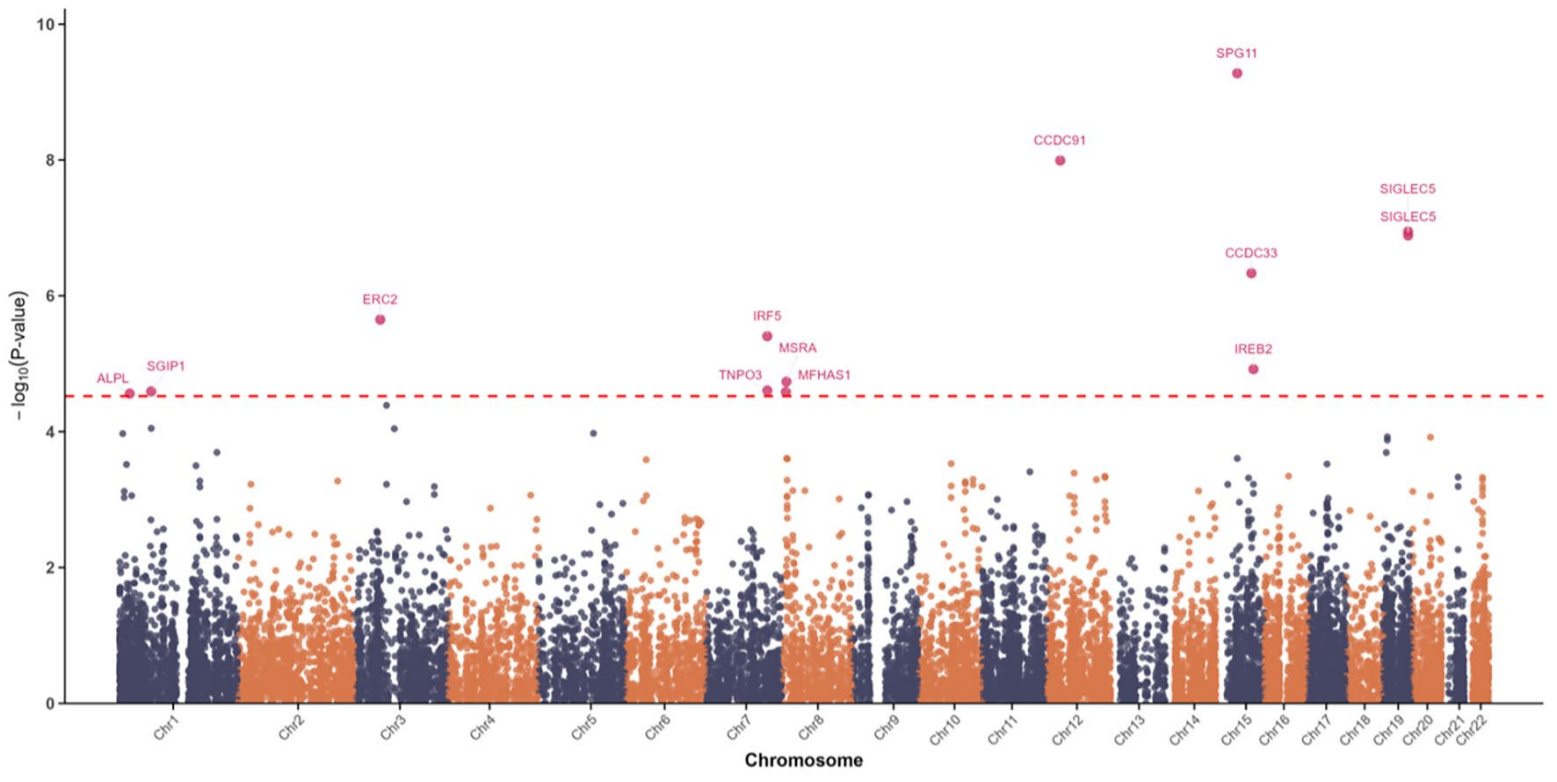
Manhattan plot of GWAS results for oral frailty from MAGMA analysis.

The oral frailty GWAS identified eight significant SNPs (P <5 × 10^–8^; Table S6, Fig 3). Most were intronic (82.7%), with others in intergenic, exonic, or UTR regions. Four independent genomic risk loci were defined (Table S7), three of which were novel: rs2705755 (SNORA77), rs150699482 (KIAA0247), and rs78975199 (SPG11) (Table S8). The remaining lead SNP, rs10843141 (near CCDC91), had prior associations with loose teeth and other traits, including waist circumference, brain volume, and chronic obstructive pulmonary disease (COPD) (Table S9). MAGMA identified 13 genes significantly associated with oral frailty (FDR <0.05), with SPG11 (Z = 6.10, P = 9.67 × 10^–6^), CCDC91 (Z = 5.61, P = 9.31 × 10^–5^), and SIGLEC5 (Z = 5.18, P = 0.00059) as top candidates (Table S10, Fig 4).

### Fine-mapping

**Fig 5:**
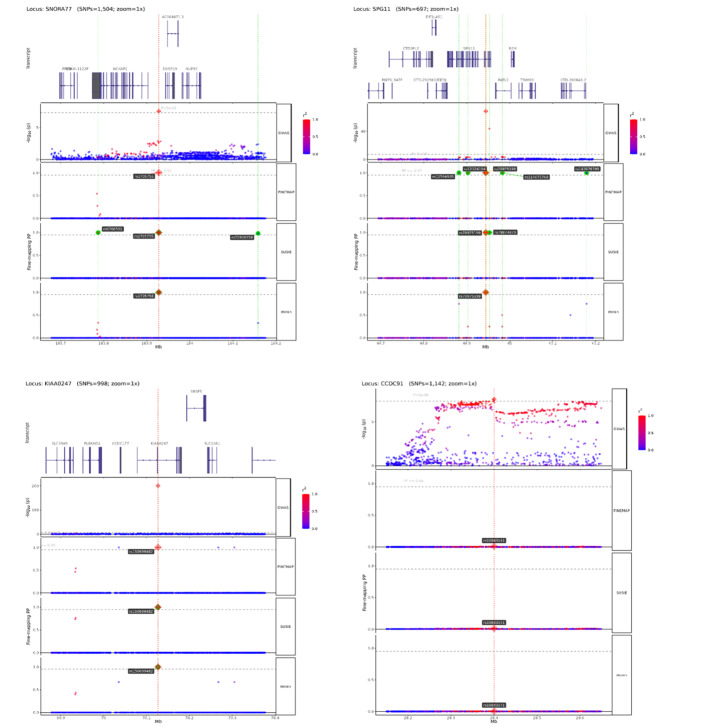
Fine-mapping results of genomic loci with strong associations (PP >0.95) identified by FINEMAP.

**Fig 6:**
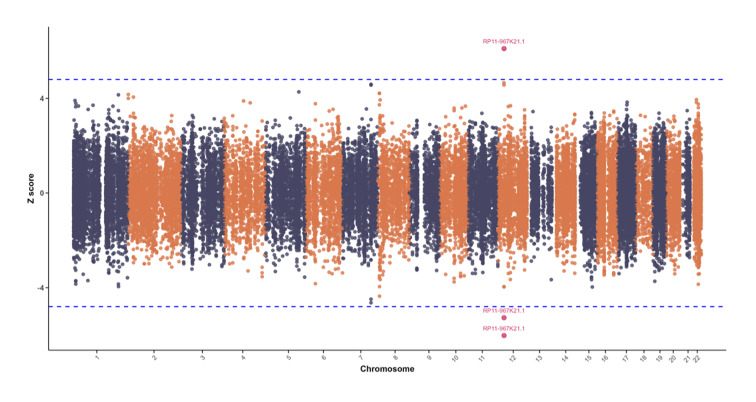
Manhattan plot of results from sCCA TWAS analysis for oral frailty. The x-axis represents chromosomes, and the y-axis displays the Z-scores.

Fine-mapping analysis identified strong association signals at three loci (mean PP >0.95 and GWAS P <5 × 10^–8^). These loci were rs150699482 near KIAA0247 (T-statistics = 48.88, GWAS P = 1.00 × 10^–200^), rs78975199 near SPG11 (T-statistics = 17.57, GWAS P = 3.97 × 10^–69^), and rs2705755 near SNORA77 (T-statistics = 5.54, GWAS P = 3.04 × 10^–8^) (Table S11, Fig 5). At the CCDC91 risk locus, however, SNP fine-mapping did not yield conclusive results. Nevertheless, TWAS analysis identified RP11-967K21.1 as significantly associated with oral frailty risk in this region, consistent across three expression panels (Table 1, Fig 6). Furthermore, FOCUS analysis further provided strong evidence supporting the causality of RP11-967K21.1 (PIP >0.8).

**Table 1:** Genetic associations with oral frailty in sCCA and FOCUS analysis

Gene	CHR	Start position	End position	Heritability squared	TWAS Z	TWAS FDR	FOCUS pip
RP11-967K21.1	12	28190737	28190738	0.3118	6.10643	1.02e-09	1
RP11-967K21.1	12	28190737	28190738	0.3639	–6.02085	1.74e-09	1
RP11-967K21.1	12	28190737	28190738	0.125	–5.2693	1.37e-07	0.998


### Pathway Enrichment and Cell-Type Enrichment Analysis

GSEA revealed significant enrichment for autoimmune disorders, including systemic lupus erythematosus, systemic sclerosis, and rheumatoid arthritis (FDR <0.05; Table S12). Additional enrichment was observed for COPD, bipolar disorder/schizophrenia, and dietary traits. Cell-type enrichment was strongest in mammary gland stromal cells (P = 0.011), with additional signals in brain endothelial (P = 0.014), liver (P = 0.018) and marrow (P = 0.021) natural killer (NK), and marrow B cells (P = 0.049) (Table S13).

### Spatially Mapping in Embryonic Tissues

The gsMap analysis revealed significant associations in the sympathetic nerve (P = 0.0040), meninges (P = 0.0044), brain (P = 0.010), jaw and tooth (P = 0.011), inner ear (P = 0.014), adipose tissue (P = 0.016), dorsal root ganglion (P = 0.017), mucosal epithelium (P = 0.022), connective tissue (P = 0.025), and choroid plexus (P = 0.026) (Table S14).

### Heritability Enrichment and Polygenic Risk Score Construction

S-LDSC revealed significant heritability enrichment in five genomic annotation categories (FDR <0.05; Table S15), particularly in evolutionarily conserved regions (enrichment factor = 10.4) and active regulatory elements (DHS and H3K4me1 marked regions), indicating that oral frailty heritability is concentrated in these functional genomic regions. We also leveraged oral frailty GWAS summary statistics for PRS construction. Analysis of PRS structure revealed significant contribution variation across chromosomes (Table S16). Chromosome 1 and chromosome 2 accounted for the largest score proportions. Conversely, chromosomes 21, 22, and 19 contributed the least amount. This observation distinctly underscores the polygenic nature of oral frailty.

## DISCUSSION

This study employed genomic SEM to elucidate the shared genetic architecture underlying oral frailty. Five key oral frailty-related phenotypes were investigated: loose teeth, Sjögren’s syndrome, dental caries, dysphagia, and chronic periodontitis. A major achievement was the identification of a latent common factor. Subsequent locus definition analyses localised four genomic regions significantly associated with oral frailty. Notably, novelty assessment and fine-mapping confirmed rs150699482, rs78975199, and rs2705755 as novel, putatively causal variants that robustly drive the association signal for oral frailty. Complementary post-GWAS analyses, including TWAS, gene-set enrichment, cell-type enrichment, partitioned heritability, and gsmap, provided multidimensional evidence supporting these findings. Collectively, these analyses elucidated the potential biological functions of oral frailty. This finding contrasts significantly with previous research, primarily focused on the genetic factors of individual dental diseases. By uncovering the common genetic underpinnings of these prevalent oral issues, this work lays a genetic foundation for a comprehensive understanding of the integrated mechanisms driving oral frailty.

Building upon the common factor model, we identified genetic variants directly associated with oral frailty. The pleiotropic nature of these loci provides a potential explanation for how a condition like periodontitis is genetically linked to broader oral dysfunction. Independent genomic risk locus definition pinpointed four lead SNPs. Subsequent GWAS-minus-locus analysis and fine-mapping highlighted rs2705755, rs150699482, and rs78975199 as novel loci with high causal probability. For example, rs150699482 may act via KIAA0247-related cellular pathways, modulating cellular stress responses and RNA splicing,^43^ while rs78975199 might interfere with SPG11-mediated cellular clearance mechanisms and disrupt inflammatory homeostasis, thereby increasing susceptibility to oral frailty.^27^ These results suggest that the genetic architecture of oral frailty involves pathways related to cellular senescence, adaptive stress responses, and regulation of inflammation. GWAS catalogue annotation revealed that rs10843141 exhibits significant pleiotropy, being associated with morphogenesis, development, and skeletal traits.^20,61^ This suggests that rs10843141 may mark deeper biological mechanisms involving development and tissue homeostasis relevant to oral frailty susceptibility. In summary, the mvGWAS identified multiple potential oral frailty susceptibility loci, whose regulatory functions and links to relevant biological pathways provide novel genetic perspectives essential for understanding the complex pathophysiology of oral frailty.

To elucidate the functional mechanisms underlying GWAS signals for oral frailty, TWAS and MAGMA were integrated to identify potential effector genes associated with the risk of oral frailty. First, at the CCDC91 locus, TWAS and FOCUS robustly pinpointed RP11-967K21.1 as a high-confidence effector transcript. MAGMA independently and strongly corroborated the association between the CCDC91 gene and oral frailty. CCDC91-derived circRNA is known to regulate muscle cells via the IGF1-PI3K/AKT pathway.^63^ Therefore, this convergence of evidence suggests that the CCDC91 locus likely influences oral frailty, potentially through the impact of RP11-967K21.1 expression on muscle-related pathways. Second, MAGMA revealed SPG11 as the most significantly associated gene with oral frailty, further supporting the results of fine-mapping. Pathogenic variants in SPG11, which encodes spatacsin, are causative for spastic paraplegia type 11 (SPG11-HSP).^44^ SPG11-HSP represents the most frequent form of complex autosomal recessive HSP, whose clinical phenotype of SPG11-HSP is characterised by progressive neurodegeneration. This condition is frequently accompanied by peripheral neuropathy and pseudobulbar involvement, the latter of which can impair swallowing and speech functions.^10^ Oral frailty involves complex neuromuscular coordination for mastication and swallowing. Consequently, SPG11 dysfunction might increase susceptibility to oral frailty by affecting these critical neural pathways. Furthermore, MAGMA analysis identified other Inflammation-related genes, such as IRF5 and SIGLEC5, linked to oral frailty risk. IRF5 is a central regulator of inflammatory responses, polarising macrophages towards a pro-inflammatory phenotype and participating in various inflammatory and autoimmune diseases.^2^ SIGLEC5, an immune receptor recognising lipid ligands, modulates innate immune responses.^51^ Notably, genetic variants in SIGLEC5 have been directly linked to the risk for periodontitis,^38^ providing a strong validation that our oral frailty factor is capturing clinically relevant signals from its constituent traits. These results suggest that inflammation and immune responses may play important roles in the pathophysiology of oral frailty. Additionally, MAGMA also identified genes pertinent to skeletal mineralisation and muscle function. The ALPL gene encodes tissue-nonspecific alkaline phosphatase, whose dysfunction leads to hypophosphatasia, a hereditary disorder characterised by impaired skeletal mineralisation and muscle weakness.^54^ The MSRA gene encodes methionine sulfoxide reductase A, crucial for protein repair and combating oxidative stress,^46^ which is a significant driver of ageing and muscle dysfunction.^22^ These findings suggest that skeletal integrity and protein homeostasis may play roles in the pathogenesis of oral frailty. In summary, the integrated TWAS and MAGMA analyses successfully linked GWAS signals to candidate effector genes and their potential underlying mechanisms. These results provide a deeper understanding of the genetic architecture of oral frailty, highlighting high-priority genes for subsequent functional validation.

Our cell-type enrichment analysis identified signals in several non-oral cell types, raising the critical question of whether these reflect true systemic ageing processes or are merely artefacts of pleiotropic gene expression. We propose that these are not mutually exclusive; rather, the observed pleiotropy is the mechanistic signature of systemic ageing. This aligns with the theory of antagonistic pleiotropy, where genes regulating fundamental biological processes confer benefits early in life but contribute to multisystem decline with age.^7^ The genes underlying our oral frailty factor are likely involved in such core processes, and their enrichment across diverse tissues is therefore not an artefact, but evidence of their systemic importance. For example, the enrichment in brain endothelial cells points to a systemic process of declining vascular integrity. The pleiotropic genes active here likely regulate the vascular health system as a whole. Their relevance to oral frailty emerges because complex functions like swallowing are exquisitely dependent on intact neural control, which in turn depends on a healthy neurovascular unit that becomes compromised by systemic vascular ageing.^66^ Similarly, the enrichment in immune cells is a direct signature of inflammaging, a systemic ageing process. The pleiotropic genes identified are likely key regulators of this systemic inflammatory tone, which has been shown to impact muscle metabolism and contribute to frailty, manifesting as conditions like sarcopenic dysphagia.^19^ Finally, the signal in mammary stromal cells likely reflects the function of a ubiquitous cell-type, the fibroblast, in systemic tissue homeostasis. Genes that regulate cellular senescence and repair capacity are inherently pleiotropic. Their age-related dysregulation leads to a systemic decline in tissue maintenance that would manifest in high-turnover tissues like the oral gingiva.^28^ In summary, our findings suggest the genetic basis of oral frailty lies in pleiotropic genes that govern the rate of systemic ageing, providing a powerful mechanistic link between oral and overall health.

To spatially localise the shared genetic signals of oral frailty during embryonic development, gsMap analysis was performed. The significant enrichment in jaw and tooth tissue was expected, as this is the primary site of diseases like periodontitis. The normal development, structural integrity, and health of the jaw and teeth are fundamental for effective mastication and clear articulation.^62^ Abnormalities in these tissues, such as tooth loss or alveolar bone lesions, are major risk factors for masticatory dysfunction ^30^nutritional intake and social activities; all of which play a vital role to ensure good general health and quality of life. Despite the rapidly ageing population, there is limited evidence regarding the risk factors that lead to masticatory dysfunction in older adults or protective factors which may help maintain masticatory ability. Furthermore, there is currently no consensus for a specific test which measures masticatory ability.\nOBJECTIVES: The objectives of this scoping review are to identify the risk and protective factors associated with masticatory dysfunction and determine the most commonly used objective measure of masticatory performance.\nDESIGN: A scoping review was performed using the PRISMA recommendations. MEDLINE (Ovid and may impair overall oral function^29^, closely aligning with the clinical manifestations of reduced masticatory ability and tooth mobility observed in oral frailty. Furthermore, significant enrichment in adipose tissue suggests its important role in the pathophysiology of oral frailty. Adipose tissue, beyond serving as an energy reservoir, functions as an active endocrine organ. Its function and distribution change with age and are central drivers of organismal ageing and age-associated diseases.^40^ Age-related adipose tissue dysfunction, such as chronic low-grade inflammation, dysregulated adipokine secretion, and ectopic fat deposition in muscle, is closely linked to sarcopenia and systemic frailty.^24,31^ These pathological changes may promote the progression of oral frailty by impairing the function of muscles involved in mastication and swallowing or by exacerbating systemic inflammation. Genetic signal enrichment in the dorsal root ganglion (DRG) is also noteworthy. The DRG and its cranial nerve counterparts, such as the trigeminal ganglion, are key nodes for transmitting somatosensory information, including tactile and proprioceptive signals from the oral region.^4^ Precise sensory input is essential for coordinating masticatory muscle movements, perceiving food properties, and initiating complex swallowing reflexes. Age-related decline in oral sensory function may lead to delayed swallowing responses,^8^ and abnormalities in oral sensation are associated with delayed pharyngeal swallowing events.^42^ Thus, genetic susceptibility in the DRG may impair the integration and processing of oral sensory information, thereby compromising the coordination and safety of mastication and swallowing, and serving as a potential contributor to oral frailty. Additionally, this study observed genetic signal enrichment in multiple embryonic tissues, including mucosal epithelium, connective tissue, muscle, and cartilage primordium. The broad association of these tissues further suggests that the pathophysiological mechanisms of oral frailty are not confined to the oral cavity but are related to more extensive systemic changes and multi-organ age-related degeneration. For example, degeneration of the muscle and skeletal systems, such as the comorbidity of sarcopenia and osteoporosis, is a major factor in frailty and functional limitation among older adults,^15^ naturally affecting mastication and swallowing functions. While changes in mucosal and connective tissues may impact oral barrier function and tissue repair. In summary, this broad tissue association reinforces the idea that the genetic risk for oral frailty, and by extension severe periodontitis, is not confined to the oral cavity but is related to systemic changes and multi-organ resilience.

While our findings are foundational, they hold several long-term clinical implications. First, by establishing that oral frailty is a complex syndrome with a shared genetic basis, our work supports a more integrated clinical approach to geriatric oral health. Rather than treating individual conditions like periodontitis or dysphagia in isolation, clinicians should view them as potential indicators of a broader, systemic vulnerability. This perspective encourages holistic assessment and management strategies for older adults. Second, the identification of specific genes and pathways (eg, those involving SPG11, CCDC91, inflammation, and neuromuscular function) provides a roadmap for future therapeutic development. These pathways could be targeted to develop novel interventions aimed not just at a single oral disease but at improving overall oral resilience and function. For instance, therapies modulating the inflammatory pathways highlighted in our study could have benefits across multiple oral frailty-related phenotypes. Finally, the PRS constructed in our study represents a first step towards genetic risk stratification. Although not yet ready for clinical use, a refined PRS for oral frailty could one day help identify high-risk individuals early in life. This would allow for targeted preventive interventions – such as intensive oral hygiene programmes, nutritional counselling, and regular functional assessments – long before irreversible functional decline occurs, thereby promoting healthier ageing.

Our study has several limitations that should be taken into consideration. A key limitation is the modest fit of our common factor model (CFI = 0.849), which fall below the conventional 0.90 cutoff. While such fixed thresholds are not universally applicable, particularly for parsimonious models or heterogeneous inputs,^35,36^ this value suggests caution. Plausible reasons for this fit include the oversimplification of a single-factor structure, significant heterogeneity among input phenotypes (eg, low h^2^ of dental caries), and methodological differences across source GWAS. Although the model proved useful for our exploratory goal of identifying shared genetic signals, its structural interpretation should be approached with caution. We recommend that future studies investigate alternative specifications, such as multi-factor or bi-factor models, and validate our findings in larger, more homogeneous data sets to confirm the robustness of the latent oral frailty construct. Then, one of the input traits, dental caries, exhibited non-significant SNP-based heritability. While our sensitivity analysis demonstrated that its inclusion did not materially alter our primary findings, future studies could benefit from incorporating GWAS summary statistics for dental caries with higher statistical power to refine the contribution of this trait to the shared genetic architecture of oral frailty. Finally, our spatial mapping analysis using gsMap relied on mouse embryonic spatial transcriptomics data. The interpretation of these findings is subject to the inherent limitations of cross-species analysis, as substantial divergence in gene regulatory landscapes can exist between mice and humans, potentially affecting the direct translatability of the results. Nevertheless, this approach provides valuable insights into the potential developmental origins of oral frailty, as many fundamental tissue patterning and organogenesis pathways are deeply conserved across mammals. Future studies incorporating human adult and developmental spatial transcriptomics data will be crucial for validating and refining these findings.

## CONCLUSION

Utilising genomic SEM, our study elucidated the shared genetic architecture of oral frailty by integrating five key oral frailty-related phenotypes, anchored by the inflammatory burden of chronic periodontitis. We identified a latent common genetic factor and discovered four independent genomic risk loci, including three novel, putatively causal variants (rs150699482, rs78975199, rs2705755). Integrative analyses with MAGMA and TWAS highlighted candidate effector genes such as SPG11, CCDC91, and RP11-967K21.1, implicating pathways related to inflammation, neuromuscular function, and tissue homeostasis, all of which are relevant to the pathogenesis of periodontal disease and its progression to broader oral dysfunction. Notably, cell-type and spatial enrichment analyses revealed that genetic risk for oral frailty extends beyond oral tissues, involving brain endothelial cells, immune cells, and multiple embryonic tissues such as jaw, tooth, adipose tissue, and DRG. Collectively, our findings reveal that oral frailty is a systemic and genetically complex condition, shaped by cross-system interactions and age-related processes. This multidimensional genetic framework advances our understanding of oral frailty and provides a foundation for future research and targeted interventions.

### Acknolwedgements

We gratefully acknowledge the original authors for providing the data sets used in this research.

#### Ethics approval and consent to participate

This study used GWAS data from prior research. Ethical approvals and consents were obtained in the original studies.

#### Data and code availability

All GWAS summary statistics used as input for this study are publicly available from their original sources, which are cited and detailed in Supplementary Table S1. The full GWAS summary statistics for the oral frailty latent factor generated by our genomic SEM analysis are available from the corresponding author upon reasonable request. All software packages used for the analyses are publicly available and have been described with version numbers in the Methods section.

#### Competing interests

The authors declare that they have no competing interests.

#### Funding

This research received no external funding.

**Table S4:** Fit indices for genomic-SEM model

chisq	df	p_chisq	AIC	CFI	SRMR
10.5207737098306	5	0.0617545335718295	30.5207737098306	0.848860897177436	0.190812762158744
Abbreviations: chisq = Chi-square test statistic, df = Degrees of freedom, p_chisq = P-value for the Chi-square test, AIC = Akaike information criterion, CFI = Comparative fit index, SRMR = standardized root mean square residual.

**Table S3:** Genetic correlations between oral frailty

	Loose teeth	Sjögren’s syndrome	Dental caries	Dysphagia	Chronic periodontitis
Loose teeth	0.0126 (0.0014)	0.1078 (0.1129)	0.296 (0.2126)	0.5384 (0.1376)	0.7192 (0.1503)
Sjögren’s syndrome	0.1078 (0.1129)	0.0052 (0.0014)	–0.1007 (0.317)	0.3898 (0.213)	0.7083 (0.2337)
Dental caries	0.296 (0.2126)	–0.1007 (0.317)	0.0011 (0.0012)	0.2942 (0.4516)	-0.3528 (0.4076)
Dysphagia	0.5384 (0.1376)	0.3898 (0.213)	0.2942 (0.4516)	0.0035 (0.0012)	0.2581 (0.273)
Chronic periodontitis	0.7192 (0.1503)	0.7083 (0.2337)	–0.3528 (0.4076)	0.2581 (0.273)	0.0029 (0.0011)


**Table S2:** SNP heritability of genomic-SEM phenotypes

Phenotype	NSNPs	h^2^ (se)	λGC	Mean chi square	Intercept se	Ratio se	h^2^ Z
Loose teeth	1154917	0.0126 (0.0014)	1.1271	1.1283	1.0152 (0.0072)	0.1183 (0.0563)	9.26
Sjögren’s syndrome	1160413	0.0052 (0.0014)	1.0587	1.0675	1.0214 (0.0079)	0.3164 (0.1167)	3.77
Dental caries	1173480	0.0011 (0.0012)	1.0141	1.0157	1.0069 (0.0063)	0.4384 (0.4019)	0.932
Dysphagia	1173501	0.0035 (0.0012)	1.0378	1.0411	1.0142 (0.006)	0.345 (0.1455)	2.84
Chronic periodontitis	1160151	0.0029 (0.0011)	1.0503	1.0465	1.0193 (0.0076)	0.4162 (0.1641)	2.52


**Table S1:** GWAS summary sources

Trait	Source	N case	N control	N
Loose teeth	GCST90044344	18545	436020	454565
Sjögren’s syndrome	Finngen R11	2981	439424	442405
Dental caries	GCST90436264	3051	398136	401187
Dysphagia	GCST90436316	6482	369275	375757
Chronic periodontitis	Finngen R11	5364	288472	293836


**Table S8:** Lead SNP identified by genomic-SEM

Novel	Lead SNP	chr	pos	p	Independent SNPs
YES	rs2705755	2	183927981	3.03762567046e-08	rs2705755
No_Loose teeth	rs10843141	12	28400187	2.76250542692e-08	rs10843141
YES	rs150699482	14	70126683	1e-200	rs150699482
YES	rs78975199	15	44944224	3.97038264379e-69	rs78975199


**Table S7:** Risk locus identified by genomic-SEM

Sequence	Locus	SNP	chr	pos	P
1	2:183927981:C:T	rs2705755	2	183927981	3.03762567046e-08
2	12:28400187:G:T	rs10843141	12	28400187	2.76250542692e-08
3	14:70126683:C:T	rs150699482	14	70126683	1e-200
4	15:44944224:A:G	rs78975199	15	44944224	3.97038264379e-69


**Table S6:** Novel SNP variants identified by genomic SEM

SNP	CHR	BP	eaf	effect_allele	other_allele	beta	se	pval	N
rs2705755	2	183927981	0.043	T	C	0.082	0.0148	3.04E-08	81973
rs10492369	12	28325118	0.305	G	A	–0.034	0.0062	4.90E-08	81973
rs11049478	12	28397760	0.304	G	A	–0.034	0.0062	3.21E-08	81973
rs10843141	12	28400187	0.304	G	T	–0.035	0.0062	2.76E-08	81973
rs11049480	12	28400314	0.305	A	G	–0.034	0.0062	4.25E-08	81973
rs150699482	14	70126683	0.023	C	T	8.448	0.1729	1.00E-200	81973
rs78975199	15	44944224	0.011	A	G	3.574	0.2034	3.97E-69	81973
rs79974470	15	44952927	0.011	G	T	–1.256	0.0899	2.30E-44	81973


**Table S5:** Genomic-SEM factor loadings and variance estimates for oral frailty traits

lhs	op	rhs	Unstandardized_Estimate	Unstandardized_SE	Standardized_Est	Standardized_SE	P value
F1	=~	Loose teeth	0.229	0.053	0.699	0.163	1.92E-05
F1	=~	Sjögren’s syndrome	0.165	0.065	0.364	0.143	0.0108
F1	=~	Dental caries	0.037	0.049	0.162	0.213	0.4465
F1	=~	Dysphagia	0.138	0.036	0.645	0.170	0.0001
F1	=~	Chronic periodontitis	0.211	0.055	0.892	0.235	0.0001
Loose teeth	~~	Loose teeth	0.055	0.025	0.512	0.231	0.0266
Sjögren’s syndrome	~~	Sjögren’s syndrome	0.178	0.054	0.867	0.262	0.0009
Dental caries	~~	Dental caries	0.051	0.056	0.974	1.063	0.3595
Dysphagia	~~	Dysphagia	0.027	0.019	0.584	0.408	0.1524
Chronic periodontitis	~~	Chronic periodontitis	0.011	0.030	0.205	0.534	0.7016
Abbreviations: lhs = left-hand side (predictor or factor), op = operator (‘=~’ denotes factor loadings, ‘~~’ denotes residual variances), rhs = right-hand side (outcome or observed variable), Unstandardized_Estimate = unstandardized factor loading or variance estimate, Unstandardised_SE = standard error of the unstandardised estimate, Standardised_Est = standardized factor loading or variance estimate, Standardised_SE = standard error of the standardised estimate.

**Table S11:** Fine-mapping of association signals

Locus	SNP	P	tstat	mean.PP	mean.CS
SPG11	rs78975199	3.97038E-69	17.57294197	1	1
KIAA0247	rs150699482	1E-200	48.87556552	1	1
SNORA77	rs2705755	3.03763E-08	5.539254139	1	1


**Table S10:** MAGMA risk gene annotation using genomic-SEM

Gene	chr	start	end	Z	P
ALPL	1	21835858	21904905	4.0332	2.7513e-05
SGIP1	1	66999066	67213982	4.0509	2.5513e-05
ERC2	3	55542336	56502391	4.588	2.2374e-06
IRF5	7	128577666	128590089	4.4688	3.9331e-06
TNPO3	7	128594948	128695198	4.0581	2.474e-05
MFHAS1	8	8640864	8751155	4.0466	2.5979e-05
MSRA	8	9911778	10286401	4.1268	1.8389e-05
CCDC91	12	28286182	28732883	5.6092	1.0164e-08
SPG11	15	44854894	44955876	6.1008	5.2763e-10
CCDC33	15	74509613	74628813	4.9062	4.6431e-07
IREB2	15	78729773	78793798	4.2237	1.2016e-05
SIGLEC5	19	52114781	52150151	5.1514	1.2928e-07
SIGLEC5	19	52115344	52150142	5.1763	1.1316e-07


**Table S9:** GWAS catalogue annotation

Lead SNP	chr	pos	p	Previous GWAS associations
rs10843141	12	28400187	2.762e-08	Waist circumference adjusted for body mass index, Hip circumference adjusted for BMI, Brain region volumes, Brain shape (segment 1), Waist circumference adjusted for body mass index, Vertex-wise cortical surface area, Vertex-wise cortical surface area, Facial morphology (segment 1), Cortical surface area, Cortical thickness (MOSTest), Whole brain restricted directional diffusion (multivariate analysis), Subcortical volume (MOSTest), Brain morphology (min-P), Subcortical volume (min-P), Chronic obstructive pulmonary disease, Brain morphology (MOSTest), Vertex-wise sulcal depth, Vertex-wise sulcal depth, Hip circumference adjusted for BMI, Brain morphology (MOSTest), Chronic obstructive pulmonary disease, Waist circumference adjusted for body mass index, Waist circumference adjusted for body mass index, Waist circumference adjusted for BMI (joint analysis main effects and physical activity interaction), Waist circumference adjusted for BMI in active individuals, Waist circumference adjusted for BMI in active individuals, Waist circumference adjusted for BMI in active individuals, Waist circumference adjusted for BMI (joint analysis main effects and physical activity interaction), Waist circumference adjusted for BMI (joint analysis main effects and physical activity interaction), Waist circumference adjusted for body mass index, Waist circumference adjusted for body mass index, Sib-shared facial trait 796; Facial segment 42; 3D morphology of the nose sides, Body fat distribution (leg fat ratio), Body fat distribution (trunk fat ratio), Body fat distribution (leg fat ratio), Body fat distribution (arm fat ratio), Body fat distribution (arm fat ratio), Body fat distribution (arm fat ratio), Body fat distribution (trunk fat ratio), Waist circumference adjusted for BMI (adjusted for smoking behaviour), Waist circumference adjusted for BMI (joint analysis main effects and smoking interaction), Waist circumference adjusted for BMI (joint analysis main effects and smoking interaction), Waist circumference adjusted for BMI (adjusted for smoking behaviour), Waist circumference adjusted for BMI (adjusted for smoking behaviour), Hip circumference adjusted for BMI, Hip circumference adjusted for BMI, Hip circumference adjusted for BMI, Waist circumference adjusted for body mass index, Waist circumference adjusted for body mass index, Waist circumference adjusted for BMI in non-smokers, Waist circumference adjusted for BMI in non-smokers, Height, Appendicular lean mass, Height, Cortical thickness, Vertex-wise cortical thickness, Narrowest width of the femoral neck, Height, Adolescent idiopathic scoliosis, Height, Hip circumference adjusted for BMI, Osteoarthritis of the hand, Waist circumference adjusted for body mass index, Height


**Table S13:** Enriched cell types in GWAS for oral frailty

Name	Coefficient	SE	P
Mammary_Gland_stromal_cell	1.36E-08	5.91E-09	0.011
Brain_Non-Myeloid_endothelial_cell	1.27E-08	5.77E-09	0.014
Liver_natural_killer_cell	1.59E-08	7.56E-09	0.018
Marrow_mature_natural_killer_cell	1.68E-08	8.28E-09	0.021
Marrow_immature_natural_killer_cell	1.70E-08	9.36E-09	0.035
Marrow_B_cell	1.50E-08	9.03E-09	0.049
Lung_natural_killer_cell	1.41E-08	8.71E-09	0.053
Liver_Kupffer_cell	1.04E-08	7.42E-09	0.080
Marrow_immature_NK_T_cell	1.30E-08	9.46E-09	0.085
Trachea_endothelial_cell	8.07E-09	5.99E-09	0.089
Lung_B_cell	1.03E-08	7.79E-09	0.093
Lung_T_cell	1.28E-08	9.69E-09	0.093
Trachea_blood_cell	9.88E-09	8.30E-09	0.117
Marrow_pre-natural_killer_cell	8.18E-09	6.90E-09	0.118
Marrow_basophil	8.86E-09	7.48E-09	0.118
Kidney_macrophage	1.08E-08	9.48E-09	0.128
Marrow_immature_T_cell	8.59E-09	7.93E-09	0.139
Marrow_regulatory_T_cell	1.04E-08	9.84E-09	0.146
Kidney_epithelial_cell_of_proximal_tubule	6.38E-09	6.11E-09	0.148
Skin_stem_cell_of_epidermis	6.64E-09	6.57E-09	0.156
Pancreas_endothelial_cell	7.41E-09	7.34E-09	0.156
Limb_Muscle_mesenchymal_stem_cell	5.50E-09	5.53E-09	0.160
Trachea_mesenchymal_cell	4.74E-09	4.81E-09	0.162
Marrow_hematopoietic_precursor_cell	5.42E-09	5.87E-09	0.178
Heart_endocardial_cell	4.59E-09	5.18E-09	0.188
Pancreas_pancreatic_stellate_cell	4.92E-09	5.56E-09	0.188
Fat_mesenchymal_stem_cell_of_adipose	4.60E-09	5.39E-09	0.197
Fat_natural_killer_cell	7.18E-09	8.53E-09	0.200
Marrow_granulocyte_monocyte_progenitor_cell	5.14E-09	6.11E-09	0.200
Fat_T_cell	5.89E-09	7.66E-09	0.221
Lung_lung_endothelial_cell	4.50E-09	6.06E-09	0.229
Marrow_naive_B_cell	5.42E-09	7.31E-09	0.229
Heart_unknown_cell_type	3.89E-09	5.46E-09	0.238
Thymus_DN1_thymic_pro-T_cell	6.00E-09	8.69E-09	0.245
Liver_endothelial_cell_of_hepatic_sinusoid	3.71E-09	5.49E-09	0.250
Spleen_macrophage	6.03E-09	9.00E-09	0.252
Marrow_late_pro-B_cell	3.52E-09	6.12E-09	0.283
Limb_Muscle_T_cell	6.04E-09	1.09E-08	0.290
Thymus_immature_T_cell	3.75E-09	6.80E-09	0.290
Mammary_Gland_endothelial_cell	4.27E-09	7.82E-09	0.292
Lung_classical_monocyte	4.55E-09	8.56E-09	0.297
Skin_keratinocyte_stem_cell	3.21E-09	6.05E-09	0.298
Marrow_macrophage	2.91E-09	6.12E-09	0.317
Pancreas_leukocyte	4.06E-09	8.58E-09	0.318
Liver_B_cell	3.62E-09	9.42E-09	0.350
Bladder_bladder_cell	1.38E-09	4.31E-09	0.375
Skin_epidermal_cell	1.47E-09	5.44E-09	0.394
Spleen_T_cell	2.11E-09	8.14E-09	0.398
Heart_myofibroblast_cell	1.40E-09	5.57E-09	0.401
Trachea_epithelial_cell	1.36E-09	5.50E-09	0.402
Lung_myeloid_cell	1.97E-09	8.54E-09	0.409
Heart_leukocyte	1.26E-09	6.22E-09	0.419
Pancreas_pancreatic_acinar_cell	1.58E-09	8.05E-09	0.422
Marrow_Slamf1-positive_multipotent_progenitor_cell	1.16E-09	6.23E-09	0.426
Marrow_immature_B_cell	9.51E-10	5.69E-09	0.434
Lung_monocyte	9.82E-10	7.39E-09	0.447
Marrow_granulocyte	3.88E-10	5.50E-09	0.472
Large_Intestine_large_intestine_goblet_cell	2.64E-10	4.90E-09	0.479
Heart_endothelial_cell	2.59E-10	6.13E-09	0.483
Fat_myeloid_cell	2.67E-10	6.36E-09	0.483
Marrow_Slamf1-negative_multipotent_progenitor_cell	8.63E-11	6.16E-09	0.494
Skin_leukocyte	–3.76E-11	8.91E-09	0.502
Spleen_B_cell	–2.76E-10	6.62E-09	0.517
Limb_Muscle_endothelial_cell	–4.60E-10	6.55E-09	0.528
Pancreas_type_B_pancreatic_cell	–3.19E-10	4.51E-09	0.528
Lung_unknown_cell_type	–5.66E-10	7.80E-09	0.529
Marrow_monocyte	–1.00E-09	6.10E-09	0.565
Brain_Myeloid_microglial_cell	–9.31E-10	5.36E-09	0.569
Limb_Muscle_lymphocyte	–1.45E-09	7.69E-09	0.575
Limb_Muscle_B_cell	–1.77E-09	7.85E-09	0.589
Limb_Muscle_macrophage	–1.70E-09	7.48E-09	0.590
Heart_fibroblast	–1.29E-09	4.95E-09	0.603
Marrow_megakaryocyte-erythroid_progenitor_cell	–1.67E-09	6.27E-09	0.605
Liver_hepatocyte	–1.61E-09	5.92E-09	0.607
Lung_leukocyte	–2.47E-09	8.93E-09	0.609
Marrow_common_lymphoid_progenitor	–1.61E-09	5.69E-09	0.611
Marrow_granulocytopoietic_cell	–1.88E-09	6.10E-09	0.621
Kidney_kidney_collecting_duct_epithelial_cell	–2.40E-09	7.01E-09	0.634
Brain_Non-Myeloid_neuron	–1.71E-09	4.26E-09	0.656
Limb_Muscle_skeletal_muscle_satellite_stem_cell	–2.99E-09	6.14E-09	0.687
Skin_basal_cell_of_epidermis	–2.75E-09	5.08E-09	0.706
Lung_stromal_cell	–2.55E-09	4.50E-09	0.715
Kidney_endothelial_cell	–3.87E-09	6.65E-09	0.719
Fat_B_cell	–4.29E-09	6.95E-09	0.732
Brain_Myeloid_macrophage	–4.10E-09	6.57E-09	0.734
Thymus_leukocyte	–5.45E-09	8.55E-09	0.738
Large_Intestine_epithelial_cell_of_large_intestine	–2.96E-09	4.52E-09	0.744
Marrow_precursor_B_cell	–3.40E-09	5.06E-09	0.749
Tongue_keratinocyte	–3.81E-09	5.42E-09	0.759
Pancreas_pancreatic_D_cell	–3.30E-09	4.29E-09	0.779
Pancreas_pancreatic_ductal_cell	–3.83E-09	4.90E-09	0.783
Pancreas_pancreatic_A_cell	–3.79E-09	4.78E-09	0.786
Mammary_Gland_luminal_epithelial_cell_of_mammary_gland	–4.01E-09	4.97E-09	0.790
Brain_Non-Myeloid_oligodendrocyte_precursor_cell	–3.50E-09	4.25E-09	0.795
Tongue_basal_cell_of_epidermis	–3.68E-09	4.39E-09	0.799
Bladder_bladder_urothelial_cell	–4.30E-09	4.68E-09	0.821
Limb_Muscle_skeletal_muscle_satellite_cell	–4.97E-09	5.34E-09	0.824
Mammary_Gland_basal_cell	–4.68E-09	4.68E-09	0.841
Brain_Non-Myeloid_astrocyte	–4.74E-09	4.37E-09	0.861
Fat_endothelial_cell	–7.24E-09	6.24E-09	0.877
Heart_professional_antigen_presenting_cell	–6.58E-09	5.49E-09	0.885
Lung_ciliated_columnar_cell_of_tracheobronchial_tree	–6.13E-09	4.84E-09	0.898
Brain_Non-Myeloid_brain_pericyte	–6.92E-09	5.45E-09	0.898
Brain_Non-Myeloid_oligodendrocyte	–5.51E-09	4.26E-09	0.902
Pancreas_pancreatic_PP_cell	–7.04E-09	5.36E-09	0.905
Heart_erythrocyte	–7.20E-09	4.90E-09	0.929
Large_Intestine_enteroendocrine_cell	–8.50E-09	5.77E-09	0.930
Kidney_leukocyte	–1.30E-08	8.27E-09	0.941
Brain_Non-Myeloid_Bergmann_glial_cell	–7.55E-09	4.75E-09	0.944
Fat_unknown_cell_type	–9.28E-09	5.61E-09	0.951
Large_Intestine_Brush_cell_of_epithelium_proper_of_large_intestine	–9.66E-09	5.83E-09	0.951
Large_Intestine_enterocyte_of_epithelium_of_large_intestine	–8.17E-09	4.28E-09	0.972
Heart_cardiac_muscle_cell	–9.89E-09	5.17E-09	0.972
Heart_smooth_muscle_cell	–1.42E-08	6.31E-09	0.988
Lung_epithelial_cell_of_lung	–2.18E-08	5.37E-09	1.000


**Table S12:** Enriched pathways

Category	GeneSet	N_genes	N_overlap	p	FDR P
GWAScatalog	Systemic lupus erythematosus	361	5	1.83E-06	0.008112
GWAScatalog	Systemic sclerosis (anti-centromere-positive)	6	2	5.64E-06	0.011635
GWAScatalog	Diffuse cutaneous systemic sclerosis	7	2	7.89E-06	0.011635
GWAScatalog	Systemic lupus erythematosus or rheumatoid arthritis	11	2	2.06E-05	0.022822
GWAScatalog	Systemic seropositive rheumatic diseases (Systemic sclerosis or systemic lupus erythematosus or rheumatoid arthritis or idiopathic inflammatory myopathies)	18	2	5.72E-05	0.042218
GWAScatalog	Limited cutaneous systemic scleroderma	18	2	5.72E-05	0.042218
GWAScatalog	Systemic lupus erythematosus and Systemic sclerosis	20	2	7.10E-05	0.044906
GWAScatalog	Chronic obstructive pulmonary disease	146	3	9.69E-05	0.045598
GWAScatalog	Bipolar disorder and schizophrenia	24	2	0.000103	0.045598
GWAScatalog	Cooked vegetable consumption	24	2	0.000103	0.045598


**Table S14:** Spatially mapping in embryonic tissues

Location	p_cauchy
Sympathetic nerve	0.003967
Meninges	0.004446
Brain	0.0102
Jaw and tooth	0.011175
Inner ear	0.014128
Adipose tissue	0.015864
Dorsal root ganglion	0.017123
Mucosal epithelium	0.021861
Connective tissue	0.025276
Choroid plexus	0.025806
Adrenal gland	0.030775
Kidney	0.031092
Muscle	0.041448
Spinal cord	0.043332
Lung	0.047174
Cartilage primordium	0.047949
Cartilage	0.052932
Submandibular gland	0.080061
Smooth muscle	0.081922
Heart	0.08729
Cavity	0.117747
GI tract	0.119757
Liver	0.380684
Epidermis	0.876807
Bone	0.984328
	

**Table S16:** Polygenic risk score and genetic contribution across chromosomal regions

Chromosome	PRS score sum
chr1	35.4233378644359
chr2	35.4934025455644
chr3	29.2153125131774
chr4	26.0842478840121
chr5	26.3220471951404
chr6	25.8036880439701
chr7	23.4015447334815
chr8	23.0334820062779
chr9	19.8204807703096
chr10	22.7255407814356
chr11	21.4750015462222
chr12	21.0488355987006
chr13	16.3493105449437
chr14	14.3512092843294
chr15	13.3590000771557
chr16	13.6062771232887
chr17	11.6436742594064
chr18	13.1375634838855
chr19	8.53356126495804
chr20	11.5463806730033
chr21	6.10960017314768
chr22	6.58549107732947
	

**Table S15:** Heritability enrichment across genomic functional and regulatory regions

Category	Enrichment	Coefficient	Coefficient_std_error	Coefficient_z-score	Enrichment FDR P
baseL2_0	1.00	–1.37E-08	9.28E-09	–1.472	
Coding_UCSC.bedL2_0	6.87	2.55E-08	6.84E-08	0.372	0.284
Coding_UCSC.extend.500.bedL2_0	1.78	–2.69E-08	2.42E-08	–1.110	0.598
Conserved_LindbladToh.bedL2_0	10.40	1.25E-07	5.31E-08	2.364	0.048
Conserved_LindbladToh.extend.500.bedL2_0	1.93	4.86E-09	8.38E-09	0.580	0.031
CTCF_Hoffman.bedL2_0	3.64	7.95E-09	7.43E-08	0.107	0.678
CTCF_Hoffman.extend.500.bedL2_0	4.20	4.56E-08	3.34E-08	1.366	0.057
DGF_ENCODE.bedL2_0	1.02	1.63E-08	3.54E-08	0.459	0.989
DGF_ENCODE.extend.500.bedL2_0	1.22	–1.60E-08	1.55E-08	–1.030	0.654
DHS_peaks_Trynka.bedL2_0	-0.41	–4.27E-08	5.11E-08	-0.835	0.678
DHS_Trynka.bedL2_0	0.14	–3.67E-08	4.16E-08	–0.881	0.697
DHS_Trynka.extend.500.bedL2_0	1.86	2.25E-08	1.77E-08	1.274	0.031
Enhancer_Andersson.bedL2_0	16.75	3.06E-07	1.75E-07	1.744	0.284
Enhancer_Andersson.extend.500.bedL2_0	0.93	–8.80E-08	5.45E-08	–1.615	0.989
Enhancer_Hoffman.bedL2_0	4.26	7.59E-08	5.30E-08	1.432	0.284
Enhancer_Hoffman.extend.500.bedL2_0	2.10	–2.84E-08	3.11E-08	–0.913	0.402
FetalDHS_Trynka.bedL2_0	1.26	1.35E-08	4.86E-08	0.277	0.979
FetalDHS_Trynka.extend.500.bedL2_0	2.61	2.45E-08	1.95E-08	1.261	0.031
H3K27ac_Hnisz.bedL2_0	1.36	3.87E-08	4.26E-08	0.907	0.255
H3K27ac_Hnisz.extend.500.bedL2_0	1.14	–4.76E-08	4.26E-08	–1.116	0.678
H3K27ac_PGC2.bedL2_0	1.66	–9.61E-09	2.88E-08	–0.334	0.284
H3K27ac_PGC2.extend.500.bedL2_0	1.68	1.63E-08	2.65E-08	0.614	0.140
H3K4me1_peaks_Trynka.bedL2_0	0.95	–2.47E-08	2.55E-08	–0.968	0.989
H3K4me1_Trynka.bedL2_0	1.88	2.56E-08	2.11E-08	1.215	0.057
H3K4me1_Trynka.extend.500.bedL2_0	1.42	–3.48E-09	1.53E-08	–0.228	0.037
H3K4me3_peaks_Trynka.bedL2_0	-0.84	–7.51E-08	5.02E-08	–1.496	0.678
H3K4me3_Trynka.bedL2_0	3.00	6.81E-08	2.94E-08	2.321	0.057
H3K4me3_Trynka.extend.500.bedL2_0	1.54	–2.39E-08	1.78E-08	–1.341	0.436
H3K9ac_peaks_Trynka.bedL2_0	0.58	–2.40E-08	6.49E-08	–0.370	0.979
H3K9ac_Trynka.bedL2_0	2.45	1.73E-08	3.66E-08	0.472	0.281
H3K9ac_Trynka.extend.500.bedL2_0	1.80	–7.21E-09	2.23E-08	–0.323	0.242
Intron_UCSC.bedL2_0	0.94	–8.19E-08	9.04E-08	–0.906	0.916
Intron_UCSC.extend.500.bedL2_0	1.14	8.13E-08	9.01E-08	0.902	0.402
PromoterFlanking_Hoffman.bedL2_0	0.38	–3.74E-08	9.90E-08	–0.378	0.979
PromoterFlanking_Hoffman.extend.500.bedL2_0	2.70	2.34E-08	3.96E-08	0.592	0.595
Promoter_UCSC.bedL2_0	1.85	6.04E-09	9.05E-08	0.067	0.754
Promoter_UCSC.extend.500.bedL2_0	1.67	–1.05E-08	7.62E-08	–0.138	0.678
Repressed_Hoffman.bedL2_0	0.89	9.95E-09	1.43E-08	0.697	0.947
Repressed_Hoffman.extend.500.bedL2_0	0.87	4.18E-09	1.29E-08	0.323	0.284
SuperEnhancer_Hnisz.bedL2_0	1.47	–3.24E-08	1.53E-07	–0.212	0.255
SuperEnhancer_Hnisz.extend.500.bedL2_0	1.49	2.94E-08	1.52E-07	0.194	0.195
TFBS_ENCODE.bedL2_0	0.72	–2.66E-08	3.05E-08	–0.873	0.974
TFBS_ENCODE.extend.500.bedL2_0	1.72	6.80E-09	1.95E-08	0.349	0.294
Transcribed_Hoffman.bedL2_0	1.03	7.53E-09	1.41E-08	0.534	0.989
Transcribed_Hoffman.extend.500.bedL2_0	1.03	4.89E-09	1.13E-08	0.431	0.974
TSS_Hoffman.bedL2_0	3.95	8.44E-08	7.99E-08	1.057	0.598
TSS_Hoffman.extend.500.bedL2_0	2.39	–3.62E-08	5.13E-08	–0.706	0.654
					
